# Walnut Flour as an Ingredient for Producing Low-Carbohydrate Bread: Physicochemical, Sensory, and Spectroscopic Characteristics

**DOI:** 10.3390/foods12173320

**Published:** 2023-09-04

**Authors:** Monika Wójcik, Dariusz Dziki, Arkadiusz Matwijczuk, Urszula Gawlik-Dziki

**Affiliations:** 1 Department of Food Engineering and Machines, University of Life Sciences in Lublin, 28 Głęboka St., 20-612 Lublin, Poland; monika.wojcik@up.lublin.pl; 2 Department of Thermal Technology and Food Process Engineering, University of Life Sciences in Lublin, 31 Głęboka St., 20-612 Lublin, Poland; 3 Department of Biophysics, Institute of Molecular Biophysics, Faculty of Environmental Biology, University of Life Sciences in Lublin, Akademicka 13, 20-950 Lublin, Poland; arkadiusz.matwijczuk@up.lublin.pl; 4 Department of Biochemistry and Food Chemistry, University of Life Sciences in Lublin, 8 Skromna St., 20-704 Lublin, Poland; urszula.gawlik@up.lublin.pl

**Keywords:** gluten-free bread, chemical composition, volume, texture, color, antioxidant properties

## Abstract

Walnut flour (WF) is a nutrient-rich source that can be used as an alternative for individuals on a gluten-free diet. This study aimed to assess the physical, chemical, and sensory changes in low-carbohydrate bread when supplemented with WF. Molecular-level changes were also examined using ATR-FTIR spectra. The bread recipe, containing buckwheat and flaxseed, was enriched with WF at levels ranging from 5% to 20%. The addition of WF resulted in increased loaf volume and decreased baking loss. Enriched bread samples showed higher protein content, while fat and available carbohydrate content decreased. Additionally, WF incorporation led to a decrease in crumb brightness and an increase in redness (from 23.1 to 25.4) and yellowness (from 23.8 to 26.7). WF also increased crumb hardness and chewiness. Moreover, the tested additives primarily influenced the intensity of FTIR spectra, indicating changes in protein, carbohydrate, and fat content, with increased band intensity observed in the protein region. We particularly recommend bread with a WF content of 15%. This type of bread is characterized by high consumer acceptance. Furthermore, compared to bread without the addition of WF, it has a higher content of phenolic compounds, protein, and fat by approximately 40%, 8%, and 4%, respectively. The antioxidant activity of this bread, determined using the ABTS and DPPH methods, is also significantly higher compared to the control bread.

## 1. Introduction

Among the population, there is an increasing intolerance to gluten—a protein present in wheat, rye, or barley—so scientists are looking for products that will be made from alternative cereals [1]. The literature documents the development of a range of gluten-free bread supplemented with alternative protein sources trying to improve its quality and nutritional value [2]. From a technological point of view, the production of gluten-free products with a quality similar to the quality of wheat-flour-based products is a challenge, because gluten plays an important role in the formation of a protein network that maintains the structure and allows the retention of gases in the dough, and as a consequence, bakery products with a higher volume are obtained [3,4].

Our previous research allowed us to develop a recipe for gluten-free bread with reduced carbohydrate content and increased protein content based on flaxseed and buckwheat flour as well as psyllium and potato fiber additives [5]. The choice of buckwheat flour was determined by its taste, because, as research shows, the composition of buckwheat bread contained more sugars, had a stronger umami taste and a more characteristic aroma [6]. According to research conducted by Lin et al. [7], wheat bread enriched with buckwheat flour received a higher score for taste, and more importantly, it had a good antioxidant effect. On the other hand, flaxseed is a valuable source of high-quality proteins, dietary fiber, vitamins, and minerals [8]. Potato fiber and psyllium were introduced into the bread recipe due to their high water holding capacity, which will consequently delay the process of its staling [9]. Bernardes et al. [10] evaluated a combination of psyllium fiber and flaxseed as a substitute for gluten and eggs in gluten-free bread. The results of the sensory evaluation showed that this type of bread, as a valuable source of protein and fiber, has an acceptance index above 70% (at the level of 1.5% and 7.5%, respectively, for psyllium and linseed flour). In addition, it has been noted that many small pores are formed in this bread recipe, minimizing the increase in firmness during storage.

In recent years, there has been growing interest in both plant and high-protein products, with an emphasis on bread as a carrier of the necessary ingredients of plant origin [11,12,13]. This type of food includes low-carbohydrate bread consumed in a ketogenic diet. The results of studies conducted by Kondo-Ando et al. [14] indicated that changing only the carbohydrate content of the staple food has a beneficial effect on glucose and lipid metabolism in T2DM patients. The demand for gluten-free ketogenic bakery products among consumers is increasing exponentially [15]; hence, scientists are looking for additives of various origins to enrich the recipe of this type of food. One such excellent plant material with a high health-promoting potential is walnuts, which can undoubtedly increase the supply of bioactive ingredients in the diet [16].

Walnuts are among the best sources of polyunsaturated fatty acids with the most preferred ratio of n-6 and n-3 fatty acids [17]. The composition of walnuts also includes vitamin E, folate, melatonin, and several polyphenols, which are considered neuroprotective compounds [18]. Furthermore, walnuts are rich in protein, magnesium, tocopherols (four forms—*α*, *β*, *γ*, and *δ*), sterols, and carotenoids [19,20].

Walnut flour (WF) is gaining more and more interest from consumers, for example, due to the fact that its regular consumption in the daily diet reduces the risk of some diseases [21]. WF is famous for its high protein and fiber content; vitamin E and minerals like potassium (7276 mg/kg), phosphorus (5157 mg/kg), iron (89 mg/kg), zinc (38 mg/kg), and copper (38 mg/kg); as well as a low quantity of carbohydrates [10,11].

Moreover, it is a valuable source of phenolic compounds [9]. WF is widely used in desserts, cakes, confectionery (various nut or chocolate cakes), ice cream, and also in many spicy dishes such as soups and sauces [22]. Chochkov et al. [21] studied the effect of the addition of WF on the quality of wheat bread. The researchers showed that the addition of this flour in the amount of up to 5% improved the porosity of the bread. On the other hand, the study conducted by Almoraie [23] concerned the effect of WF on the physical and sensory properties of wheat bread. Enhancing wheat flour with WF led to a decrease in the loaf volume, while the sensory analysis showed that the addition of WF up to 30% had a positive effect on the taste, aroma, and texture of the bread. However, there are no reports in the literature on other types of bread, including low-carbohydrate and gluten-free bread enriched with WF, which can be considered functional bread. Almoraie [23] found that a 50% replacement of wheat flour with WF decreased the carbohydrate content in bread from 68% to 18%, whereas Chochkov et al. [21] did not investigate this matter. Hence, in this work, analyses of the physical and antioxidant properties of high-protein and low-carbohydrate WF-enriched bread were performed. Moreover, changes in molecular properties of bread samples were determined through the spectroscopic analysis of FTIR bands.

## 2. Materials and Methods

### 2.1. Materials

The following agents (purchased from Sigma-Aldrich Poznań, Poland) were used for this study: sodium salicylate, gallic acid, ferrozine (3-(2-pyridyl)-5,6-bis-(4-phenyl-sulfonic acid)-1,2,4-triazone), and ABTS (2,2′-azino-bis-(3-ethylbenzothiazoline-6-sulfonic acid)) in analytical purity.

White buckwheat flour (Helcom, Kraków, Poland), flaxseed flour (Bio Planet, Gniezno, Poland), and partially defatted walnut flour from manufacturer OlVita, Poland (48% protein, 16% fat, 27% carbohydrates—according to the information supplied by the manufacturer), and psyllium (Dimica, Koszyce, Slovakia), potato fiber (Spiegel Hauer, Pirna, Germany), guar gum (Bio Planet, Gniezno Poland), dried yeast (LeSaffre, Maisons-Alfort, France), and Himalayan salt were used in the bread recipe.

### 2.2. Bread-Making Procedure

Samples of a mixture of flour (buckwheat and flaxseed 1:1) with an appropriate percentage of WF (5, 10, 15, and 20% in relation to the weight of basic flours, replacing buckwheat and flaxseed flour) were sieved, weighed, and combined with other technological ingredients such as psyllium, dried yeast, guar gum, and salt. To all samples, 130 mL of water 20 °C was added directly from the tap at room temperature. The control bread dough was made of 50 g of white buckwheat flour, 50 g of flaxseed flour, 4 g psyllium, 2 g of salt, 3 g of guar gum, 1 g of dried yeast, and 130 mL of water. Subsequent trials with the appropriate addition of WF (5, 10, 15, and 20 g) had a reduced amount of base flours (white buckwheat and flaxseed flour), so thatthe weight of all flours used was 100 g. The dough mixing process took 5 min, where the dough was initially mixed at 100 rpm (approx. 2 min) and then increased to 200 rpm and mixed for another 3 min, and then 120 g pieces of dough were formed by hand and put into the loaf tins (96 × 60 mm top; 80 × 50 mm bottom; 40 mm deep). The dough fermentation process was successively carried out at 30 °C and 75–88% humidity for 1 h. After this time, the samples were moistened with water and placed in a baking oven (Sadkiewicz Instruments, Bydgoszcz, Poland) heated to a temperature of 210 °C. The baking process took 20 min. The obtained bread samples, after cooling down to room temperature, were further analyzed.

### 2.3. Analysis of Basic Properties of Bread

The basic composition of bread was evaluated according to AACC standards available online [24]. The contents of moisture (Method 44-15.02), fat (Method 30-10.01), ash (Method 08-01.01), protein (PR), and dietary fiber content (TDF) (Method 32-05.01) were determined. The content of available carbohydrates and total carbohydrates was also determined [25].

The volume of obtained loaves of bread was determined using the millet seeds displacement method [26] converting to 100 g of bread. The pH of the bread crumb was also measured using the pH meter 206-ph2 (Testo, Pruszków, Poland). The baking loss was calculated and is expressed as a percentage (%) by taking the difference between the weight of the dough before and after baking, and then dividing it by the weight of the dough before baking.

### 2.4. Color Measurements of Bread

The measurement of the color of the bread crumb was carried out using a CR 30-16 colorimeter (Precise Color Reader, 4Wave, Tychy, Poland). This device works based on the CIE L*a*b* color system, where the L* value indicates lightness of color (0–100), a* denotes the red/green value (range of −150 to +100), and b* defines the blue/yellow (range of −100 to +150). The total color difference (ΔE) was calculated between the control low-carbohydrate bread and the WF-enriched bread [27]. The head of the measuring colorimeter was placed directly on the central part of the crumb of a slice of bread immediately after it was cut. The measurements were repeated three times.

### 2.5. Sensory Evaluation of Bread

The sensory evaluation was carried out 24 h after baking by forty untrained panelists (25–60 years old). Round bread crumb samples with a diameter of 28 mm were cut from a slice of bread of 10 mm thickness, coded, and submitted for evaluation in a closed odor-free room. The sensory attributes such as texture, color, taste, smell, and overall acceptability of bread were assessed. The degrees of liking bread with varying amounts of WF were based on a nine-point hedonic scale [28]. The consumers were informed about the objective of this study before the test and provided their approval according to the ethical committee of the University Life Sciences in Lublin, Poland.

### 2.6. Texture Parameters of Bread Crumb

The texture parameters of the bread crumb were tested using the TPA test after 24 h and 48 h of baking. Using a cutter with a diameter of 28 mm, samples were cut from the center of a slice (15 mm thick). Later, the samples prepared in this way were placed on the bottom plate of the ZWICK Z020/TN2S (Zwick Roell Group, Ulm, Germany) testing machine and were compressed twice to 60% of its thickness, at a speed of 20 mm·s^−1^, using a mandrel with a diameter of 25 mm. The head was used with a load force range of up to 100 N. The following parameters were determined [29] using the testxpret Simulation V.7.1 software: hardness, cohesiveness, springiness, and chewiness. The measurements were carried out in eight repetitions for each analyzed sample of bread.

### 2.7. Total Phenolic Content and Antioxidant Properties

The methanolic extract was prepared for the determination of total phenolics and antioxidant activity of flour and bread samples according to the procedure described by Różyło et al. [30]. Total phenolics content was determined based on Folin–Ciocalteu’s method with slight modifications [31]. Moreover, radical-scavenging activity against stable DPPH and ABTS radicals was determined as described by Brand-Williams et al. [32] and Re et al. [33]. The mentioned assays and used modifications were described in detail by Dziki et al. [34]

### 2.8. Infrared Spectra Measurements

FTIR spectra were measured for the analyzed samples of flour and flour with the addition of WF using an IRSprit spectrometer by Shimatzu (Osaka, Japan). The measurements were conducted with the use of an ATR (Attenuated Total Reflection) attachment comprising a ZnSe crystal with suitable geometry (in our case—truncated at 45°). This facilitated 20-fold internal reflection of the laser beam in the spectrometer. A total of 24 scans were registered during every measurement. The software automatically averaged the obtained results. Prior to and after each measurement, the crystal was thoroughly cleaned with ultrapure solvents purchased from Sigma-Aldrich (Hamburg, Germany). For exactly 1 h prior to and during each measurement, neutral N2 atmosphere was maintained inside the measurement chamber. The spectra were registered within the spectral range from 400 to 3800 cm^−1^ at a resolution of 2 cm^−1^. The measurements were conducted at the Laboratories of the Department of Biophysics, University of Life Sciences in Lublin. Prior to analysis, all the spectra were processed using GramsAI software (ver. 93) from ThermoGalactic Industries (Watham, MA, USA). The spectra were measured at room temperature.

### 2.9. Statistical Analyses

The experiments were conducted in triplicate unless mentioned otherwise. The obtained results were subjected to statistical analysis using Statistica 12.0 program (StatSoft, Cracow, Poland). One-way analysis of variance (ANOVA) was performed, and Tukey’s test was utilized to compare the average values at a significance level of α = 0.05.

## 3. Results and Discussion

### 3.1. Basic Properties of Bread

Table 1 presents the fundamental characteristics of low-carbohydrate bread supplemented with walnut flour (WF). Bread loaves with the addition of WF exhibited a lower baking loss, measuring 13.6%, compared to the control bread, which experienced a loss of approximately 15%. Furthermore, an increase in the percentage of WF resulted in larger loaf volumes, indicating that WF strengthened the dough structure. It is probable that the components of WF interacted with the food matrix, enhancing the dough’s structure and further increasing the hardness of the bread crumb. The protein and fiber found in WF exhibited high water absorption and swelling abilities [23], contributing to the reinforcement of the crumb’s structure, its capacity to retain fermentation gases, and water retention. The bread with 20% WF displayed the highest volume, while the control bread without this additive had the lowest volume. This finding is in contrast to a previous study on wheat bread, which found that adding WF above 5% led to a decrease in bread volume compared to the control bread [21]. Conversely, the results from Almoraie [23] suggested that supplementing with 20% WF could provide favorable weight and volume characteristics for bread compared to wheat bread. Regarding the crumb’s pH, a slight decrease was observed from 5.3 to 5.2 for bread fortified with WF. Bread loaves supplemented with WF above 5% had a similar pH level of 5.2 in the crumb. This suggests that the WF did not chemically react with the components or impact the acid–base balance whet incorporation into the proposed bread recipe.

The addition of walnut flour (WF) had an impact on the chemical composition of the bread loaves produced. As anticipated, the protein content significantly increased from 22.6 g/100 g DM (dry mass) in the sample without WF to 34.7 g/100 g DM in the bread with 20% WF, as shown in Table 2. Simultaneously, there was an increase in fat and a decrease in carbohydrate content from 3.26 g to 7.25 g/100 g DM and from 22.35 g to 7.16 g/100 g DM, respectively, comparing the control bread to the bread with 20% WF. These findings align with a study conducted by Almoraie [23], which also demonstrated that the addition of WF to wheat bread increased protein content and decreased carbohydrate content. Moreover, Offia-Olua [35] reported that incorporating walnut flour in wheat-walnut flour blends increased the protein content from 12.7% (for control wheat flour) to 25.5% (for 50% WF). In our study, the control bread exhibited higher soluble fiber (32.42 g/100 g DM) and total fiber contents (46.2 g/100 g DM) compared to other bread samples. However, there were no significant differences observed in total fiber content for bread with 5% and 10% WF. This could be attributed to the presence of fiber-rich flaxseed flour and psyllium in the bread recipe. Furthermore, the results indicated that the addition of WF led to a slight decrease in soluble fiber and, consequently, total fiber, reaching 31.1 g and 45.2 g/100 g DM, respectively, in the sample with 20% WF. Conversely, the levels of insoluble fiber remained similar across all analyzed bread samples, ranging from 14 g (control bread) to 14.18 g/100 g DM (bread with 10% and 20% WF). Generally, the presence of total dietary fiber has health benefits, including reducing the risk of chronic diseases such as type 2 diabetes, cardiovascular disease, and cancer [36]. Awofadeju et al. [37] reported that supplementing wheat flour with various levels of walnut flour improved crude fiber content. The ash content ranged from 5.3 g (control bread) to 5.6 g/100 g DM (bread with 20% WF). The higher ash content resulting from WF supplementation suggests an increased mineral content in the bread samples, as walnuts are rich in minerals, particularly potassium, magnesium, and calcium [18].

### 3.2. Crumb Color

The addition of walnut flour (WF) resulted in a change in the color of the bread crumb, making it browner and consequently reducing the lightness values. The most significant decrease in crumb lightness was observed in bread prepared with 20% WF, where the lightness value decreased from 39.5 to 32.3. Additionally, the redness and yellowness of the crumb also increased with the addition of WF. As a result, the total color difference (∆E) increased from 4.2 to 9.8 (Table 3). If ∆E is in the range of 0–2.0, it would not be possible to recognize the difference between the samples. If the range of Δ*E* is between 2.0 and 3.5, it would be possible for an inexperienced observer to recognize it, and with a value above 3.5, the difference in color would become clear [31]. Taking the above into account, it is evident that all levels of addition of WF caused a visible change in color compared to the control bread without WF. This finding is consistent with the research conducted by Pycia and Ivanišová [16], who also reported a decrease in lightness and an increase in redness when enriching wheat bread with walnuts. The researchers mentioned that the change in crumb color resulting from nut enrichment, such as walnuts or hazelnuts, can be attributed to the presence of carotenoid pigments and flavonoids in the nuts themselves. These natural compounds contribute to the altered color of the bread crumb.

### 3.3. Sensory Evaluation Results

**Figure 1:**
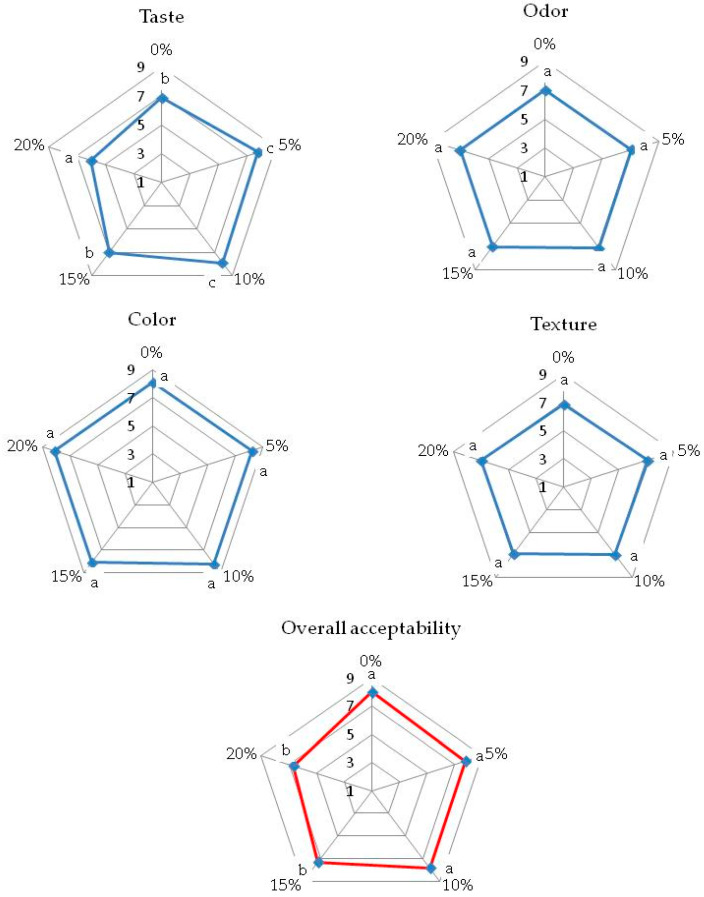
Results of the sensory evaluation of low-carbohydrate bread supplemented with walnut flour. Values designated in different letters are significantly different (α = 0.05).

**Figure 2:**
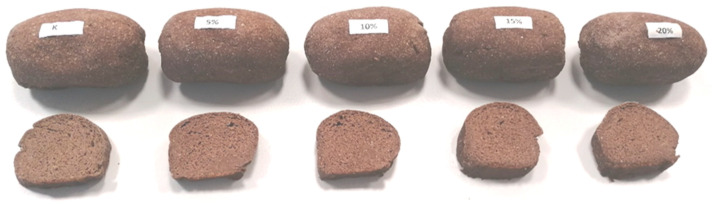
The loaves of low-carbohydrate bread enriched with the addition of WF and their cross-section.

In all the bread samples examined, the attributes that received the highest ratings were color and overall acceptability, indicating that bread supplemented with walnut flour (WF) was visually appealing (Figure 1 and Figure 2). The texture and odor of the low-carbohydrate bread crumb also received high ratings. Bread samples with up to 10% of WF addition obtained the highest scores for taste (7.9), while the sample with 20% of WF addition received the lowest score (6.0). The bread with 20% WF had a slightly bitter taste, which may have led to lower scores for this attribute. The control sample, along with samples with 5% and 10% WF additions, received the highest marks for overall acceptability (8.0, 7.9, and 7.8, respectively). Enriching the bread with up to 20% WF did not cause significant changes in the quality of the analyzed low-carbohydrate bread crumb. A study conducted by Chochkov et al. [21] demonstrated that wheat bread with up to 5% of WF addition achieved the best results in sensory evaluation compared to the control bread. However, Almoraie [23] suggested that supplementing wheat bread with 30% WF is recommended for improving both sensory attributes and nutrition. Overall, the addition of WF had positive effects on the visual appeal, texture, and odor of the bread, but the taste ratings varied depending on the percentage of WF added. The control sample and those with 5% and 10% WF additions were considered highly acceptable by the evaluators. However, breads with 15 and 20% of WF were also acceptable. The analysis of the correlation between the sensory evaluation and instrumental measurement of texture did not show any significant dependencies, which means that the increased hardness of the WF-enriched bread had no significant influence on bread acceptability.

In the case of wheat bread, enriching it with various plant additives often leads to a decrease in consumer acceptability, mainly due to the weakening of gluten structure, resulting in a decrease in volume and deterioration of texture characteristics, leading to a decline in consumer acceptability of the bread [25,38,39]. The situation is somewhat different to gluten-free bread, where such products are considered to be poorly acceptable. Therefore, the appropriate use of various enriching additives in gluten-free bread often has a beneficial impact on their sensory evaluation. This trend has been observed, for example, when adding okra flour to gluten-free bread [40], extracts from flaxseed by-product [41], or citrus fiber [42]. However, there are also several studies in which the applied additives improve the nutritional value of bread but cause a decrease in consumer acceptability of the products [43,44,45].

### 3.4. Texture Parameter Results

Table 4 presents the texture parameters of the bread crumb enriched with walnut flour (WF) after 24 and 48 h of storage. An increase in the hardness value of the bread crumb was observed with an increasing proportion of WF. The samples with a 20% WF content showed the highest hardness after 24 h of storage (43.6 N) and the samples with 15% and 20% WF showed the highest hardness after 48 h of storage (42.7 N and 54.6 N, respectively). Typically, the hardness of the crumb increases when various plant-based ingredients are incorporated into the bread recipe due to a decrease in loaf volume.

However, in this study, the addition of WF resulted in an increase in bread volume, indicating that this additive strengthens the dough structure and leads to an increase in crumb hardness. Interestingly, the results found by Pycia and Ivanišová [16] showed that WF had no significant influence on crumb hardness when wheat flour was replaced with WF at levels of 1–9%. Furthermore, the values of bread crumb hardness were significantly lower and did not exceed 13 N. This is mainly due to the fact that wheat bread, compared to gluten bread, has a much larger volume and lower crumb density, which directly translates into lower hardness. Other authors [46] analyzed the texture of gluten-free bread with reduced carbohydrate content, enriched with poppy seed flour, and found significantly higher crumb hardness, which did not exceed 25 N, after both 24 and 48 h of storage. Similar values for gluten-free bread were observed by Wójcik et al. [5], when enriching it with pea protein. From the above, it can be concluded that the type of ingredients used in bread production largely determines the textural characteristics of the crumb.

### 3.5. Total Phenolic Content and Antioxidant Activity

**Figure 3:**
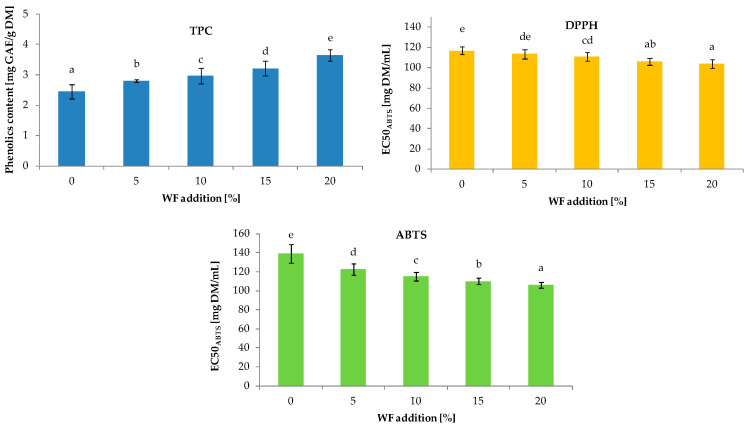
Total phenolics content (TPC) and antioxidant activity (against ABTS and DPPH radicals) of bread samples with walnut flour (WF). Histograms marked with different letters are significantly (α = 0.05) different.

Enriching cereal products with various plant raw materials is a current trend. Such activities increase the nutritional and nutraceutical value of food. According to the data presented in Figure 3, WF contained approximately twofold higher total phenolics than control bread. Consequently, replacing basic flours with WF increased the phenolic content in the bread samples. The content of TPC in the enriched bread ranged from 2.45 to 3.65 for products with 5% and 20% of WF, respectively. The coefficient of correlation between the level of WF and TPC was 0.988 (*p* < 0.05). The increased phenolic content in the bread recipe resulted in higher antioxidant activity (AA) in the enriched bread. In both the ABTS and DPPH assays, WF decreased the EC50 index, indicating an increase in the antioxidant capacity of the enriched samples. However, in the case of ABTS antiradical activity, WF only slightly increased the AA of the bread, and the value of this index decreased with the addition of WF from 116.73 mg DM/mL (control bread) to 103.71 mg DM/mL (bread with 20% WF). On the other hand, in the case of the DPPH assay, the values changed within a wider range, from 139.91 mg DM/mL to 106.17 mg DM/mL, respectively. The correlation coefficients between WF content in bread and ABTS and DPPH were statistically significant (*p* < 0.05) and resulted in r = 0.991 and 0.955, respectively. Pycia and Ivanišová [16] obtained similar results when they incorporated WF into wheat bread. However, they noted higher levels of TPC in their enriched bread samples compared to the levels of phenolics in this study. This difference may result from the different methods of WF preparation. According to literature data from Santos et al. [47], the content of TPC in WF strongly depends on thermal treatment.

### 3.6. FTIR Spectroscopic Analysis

**Figure 4:**
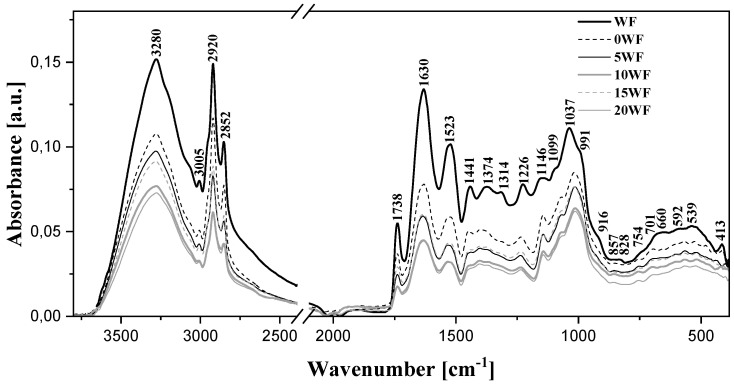
FTIR spectra for walnut flour (WF) and bread samples in the range of 450–3800 cm^−^^1^, measured at room temperature; 0 WF, 5 WF, 10 WF, 15 WF, 20 WF-bread with 0, 5, 10, 15, and 20% of WF, respectively.

In order to analyze the samples of flour and flour with the addition of WF at the molecular level, FTIR spectroscopy measurements were performed. The processed spectra are presented in Figure 4.

As follows from the data available in the literature [48,49,50,51,52,53], the characteristic vibrations with the maximum at ~3280 cm^−^^1^ are associated with the stretching vibrations of –OH groups characteristic of starch molecules, the additives used, as well as water. With the increasing content of WF, a significant decrease in the intensity of the vibrations in this range was observed. This may evidence a strong influence of vibrations originating from intermolecular hydrogen bonds with the increasing presence of the additive. The region with the maxima at ~3005, 2920, and 2852 cm^−^^1^ corresponded to stretching vibrations characteristic of -C–H groups in -CH2 groupings [48,49]. With the increasing presence of the additive, a significant decrease in their intensity was observed. This fact could be associated with a change in the content of carbohydrates in the resulting product. In turn, the deformation vibrations of hydroxyl groups are represented by the band with the maximum at approx. ~1630 cm^−^^1^. However, in the case of this type of food sample, the band with the maximum at approx. 1630 cm^−^^1^ corresponds to the very characteristic vibrations of the Amide I structure, i.e., mainly stretching vibrations of the C=O and C-N groups and deformation vibrations of the N-H group. In the relevant samples, it constitutes the primary indication of the second-tier protein structure [48]. The characteristic band with the maximum at ~1738 cm^−^^1^ reflects the stretching vibrations of the carbonyl group [42]. Changes in its intensity evidence a higher content of protein in the resulting product, and therefore a change in its quality. The characteristic bands with the maximum at ~1523 cm^−^^1^ belong to the vibrations of the Amide II structure [50,53], i.e., correspond to the deformation vibrations of the N-H group, stretching vibrations of the C-N group, and stretching of the carbonyl group. The vibrations with the maximum at ~1441 cm^−^^1^ are also characteristic vibrations of protein structures [52]. They can be additionally enhanced by the contribution of deformation vibrations originating from -CH2 as well as ν(-COO-) groups [50,53]. Next, we can look at the bands with the maximum at ~1226 cm^−^^1^, also characteristic of proteins, which correspond to vibrations of the Amide III group, i.e., mainly stretching vibrations of the C-N, deformation of the N-H, and stretching of the C-C groups [50]. The maxima at ~1146, 1099, and 1037 and enhancement at 991 cm^−^^1^ correspond to the characteristic C-O stretching vibrations and stretching of the C-C groups and, above all, the ν(C-O-C) structure. It is also noteworthy that the ratio intensity for bands located at 1037 and 991 cm^−1^, which indicates the degree of crystallinity, did not change significantly in our samples.

Vibrations in this spectral range are characteristic of the crystalline and amorphous region of starch [49,53] as well as the carbohydrate additives. The vibrations with the maxima at ~1146 and 1099 are fairly characteristic of polysaccharide molecules [53] and usually have significantly higher intensity. This shows that the additive affected the intensity of molecular interactions in the structure of starch. In turn, changes in just the intensity of bands within the range from 950 to 480 cm^−^^1^ also correspond to vibrations in the bonds of the sugar fractions present in the structure of starch [51]. Meanwhile, changes in both the intensity and shape of the bands also reflect the formation of hydrogen bonds between starch units. Moreover, the region may be enhanced by vibrations on the α-1,4-glycoside and α-1,6-glycoside bonds between the primary mers of starch [53]. As such, the short-range order of the double or single helical amylose and amylopectin, located within the amorphous or crystalline lamellar regions of starch, and complexes of the amylose-amylopectin helix, was slightly reorganized after the introduction of the additive. The microstructures and conformations present in starch may change fairly significantly with the introduction of particular additives or changes in the sample’s moisture content, as clearly observable in our studies.

## 4. Conclusions

Replacing buckwheat and linseed flour with WF resulted in low-carbohydrate bread with a higher volume and lower baking loss, while still maintaining the desired sensory quality for consumers. Additionally, the protein content increased, while the fat and available carbohydrates content decreased in the enriched bread samples. The bread enriched with WF exhibited a darker, redder, and more yellow crumb compared to the control bread. Furthermore, the incorporation of WF notably increased the antioxidant activity of the bread. However, it also led to an increase in crumb hardness and chewiness. The analysis of FTIR spectra clearly indicated that the primary structural skeleton of starch, consisting of amylase and/or amylopectin, was preserved in the end products. We specifically suggest opting for bread containing 15% WF content. This bread variety is recognized for its high sensory acceptance by consumers. Moreover, in comparison to bread lacking WF incorporation, it possesses elevated levels of phenolic compounds, protein, and fat. Future studies on this type of bread should focus on determining the shelf life and optimal storage conditions of such kinds of products.

## Figures and Tables

**Table 1:** Basic properties of low-carbohydrate bread supplemented with walnut flour.

Properties of Bread	Addition of WF (%)				
	0	5	10	15	20
Baking loss (%)	14.8 ± 0.45 ^b^	13.1 ± 0.48 ^a^	13.3 ± 0.33 ^a^	13.6 ± 0.49 ^a^	13.6 ± 0.49 ^a^
Volume of 100 g of bread (cm^3^)	147.5 ± 1.12 ^a^	153.6 ± 0.93 ^b^	158.2 ± 0.44 ^c^	163.1 ± 0.67 ^d^	164.5 ± 0.47 ^d^
pH (−)	5.3 ± 0.03 ^a^	5.3 ± 0.03 ^a^	5.2 ± 0.03 ^b^	5.2 ± 0.03 ^b^	5.2 ± 0.03 ^b^

Values in the same line marked with different letters are significantly different (α = 0.05), WF—walnut flour.

**Table 2:** Chemical composition (% DM) of low-carbohydrate bread enriched with walnut flour.

Addition of WF (%)	Protein	Fat	Ash	Soluble Fiber	Insoluble Fiber	Total Fiber	AC	TC
0	22.65 ± 2.53 ^a^	3.26 ± 0.03 ^e^	5.33 ± 0.03 ^a^	32.42 ± 0.08 ^a^	14.00 ± 0.04 ^b^	46.41 ± 0.06 ^a^	22.35 ± 2.40 ^c^	68.76 ± 2.12 ^e^
5	25.78 ± 1.27 ^ab^	4.18 ± 0.02 ^d^	5.35 ± 0.04 ^a^	32.29 ± 0.07 ^b^	14.04 ± 0.03 ^bc^	46.33 ± 0.03 ^a^	18.36 ± 1.21 ^bc^	64.69 ± 1.18 ^d^
10	28.39 ± 2.20 ^bc^	5.24 ± 0.11 ^c^	5.41 ± 0.04 ^ab^	32.01 ± 0.05 ^b^	14.18 ± 0.07 ^a^	46.19 ± 0.11 ^a^	14.77 ± 1.95 ^abc^	60.96 ± 1.84 ^c^
15	30.39 ± 0.48 ^cd^	6.46 ± 0.07 ^b^	5.48 ± 0.04 ^b^	31.62 ± 0.17 ^c^	14.13 ± 0.05 ^ac^	45.76 ± 0.12 ^b^	11.91 ± 0.40 ^ab^	54.67 ± 0.38 ^ab^
20	34.71 ± 0.69 ^d^	7.25 ± 0.09 ^a^	5.60 ± 0.02 ^c^	31.10 ± 0.07 ^d^	14.18 ± 0.02 ^a^	45.28 ± 0.06 ^c^	7.16 ± 0.73 ^a^	52.44 ± 0.69 ^a^

Values in the same column marked with different letters are significantly different (α = 0.05), AC—available carbohydrates, TC—total carbohydrates.

**Table 3:** Results of the color parameter of low-carbohydrate bread supplemented with walnut flour.

Addition of WF (%)	L*	a*	b*	C*	h°	ΔE
WF	49.1 ± 0.22	8.6 ± 0.22	27.54 ± 0.12	28.8 ± 0.15	72.7 ± 0.10	—
0	39.5 ± 0.12 ^a^	5.7 ± 0.14 ^a^	23.1 ± 0.15 ^a^	23.8 ± 0.26 ^a^	76.1 ± 0.12 ^a^	—
5	37.2 ± 0.10 ^b^	8.2 ± 0.11 ^b^	22.7 ± 0.12 ^a^	24.3 ± 0.22 ^a^	70.3 ± 0.23 ^c^	4.2 ± 0.07 ^a^
10	35.7 ± 0.12 ^c^	8.5 ± 0.21 ^b^	25.4 ± 0.25 ^b^	26.7 ± 0.20 ^b^	71.5 ± 0.15 ^b^	6.5 ± 0.02 ^a^
15	34.9 ± 0.08 ^c^	8.5 ± 0.08 ^b^	25.2 ± 0.18 ^b^	26.6 ± 0.24 ^b^	71.4 ± 0.20 ^b^	7.3 ± 0.23 ^a^
20	32.3 ± 0.18 ^d^	8.4 ± 0.12 ^b^	25.4 ± 0.15 ^b^	26.7 ± 0.19 ^b^	71.7 ± 0.12 ^b^	9.8 ±0.13 ^a^

Values in the same column marked with different letters are significantly different (α = 0.05).

**Table 4:** Texture parameters after 24 h and 48 h of storage of low-carbohydrate bread crumb with addition of WF.

Addition of WF (%)	Hardness (N)	After 24 h Cohesiveness (-)	Springiness (-)	Chewiness (N)
0	28.6 ± 0.25 ^a^	0.26 ± 0.03 ^a^	0.67 ± 0.02 ^a^	4.9 ± 0.12 ^a^
5	29.4 ± 0.58 ^a^	0.30 ± 0.02 ^ab^	0.72 ± 0.01 ^b^	6.2 ± 0.22 ^a^
10	33.1 ± 0.55 ^b^	0.31 ± 0.04 ^b^	0.76 ± 0.03 ^b^	6.4 ±0.31 ^a^
15	33.1 ± 0.38 ^b^	0.29 ± 0.01 ^ab^	0.70 ± 0.01 ^ab^	6.8 ± 0.21 ^ab^
20	43.6 ± 0.61 ^c^	0.25 ± 0.05 ^a^	0.71 ± 0.02 ^ab^	7.2 ± 0.15 ^b^
After 48 h				
0	34.6 ± 0.45 ^a^	0.26 ± 0.02 ^ab^	0.59 ± 0.04 ^a^	5.3 ± 0.13 ^a^
5	36.4 ± 0.36 ^a^	0.31 ± 0.03 ^b^	0.70 ± 0.03 ^b^	7.4 ± 0.21 ^b^
10	37.7 ± 0.24 ^a^	0.26 ± 0.02 ^ab^	0.71 ± 0.02 ^b^	7.2 ± 0.12 ^b^
15	42.7 ± 0.42 ^b^	0.23 ± 0.02 ^a^	0.61 ± 0.02 ^a^	7.2 ± 0.11 ^b^
20	54.6 ± 0.26 ^c^	0.21 ± 0.06 ^a^	0.60 ± 0.01 ^a^	7.1 ± 0.09 ^b^

Values in the same column marked with different letters are significantly (α = 0.05) different.

## Data Availability

The data presented in this study are available on request from the corresponding author.
